# When the Eyes No Longer Lead: Familiarity and Length Effects on Eye-Voice Span

**DOI:** 10.3389/fpsyg.2016.01720

**Published:** 2016-11-02

**Authors:** Susana Silva, Alexandra Reis, Luís Casaca, Karl M. Petersson, Luís Faísca

**Affiliations:** ^1^Center for Psychology at the University of Porto, University of PortoPorto, Portugal; ^2^Centre for Biomedical Research, University of AlgarveFaro, Portugal; ^3^Neurobiology of Language, Max Planck Institute for PsycholinguisticsNijmegen, Netherlands; ^4^Language in interaction, Donders Institute for Brain, Cognition and BehaviourNijmegen, Netherlands

**Keywords:** eye-voice span, eye-tracking, reading aloud, dual-route, sublexical processing

## Abstract

During oral reading, the eyes tend to be ahead of the voice (eye-voice span, EVS). It has been hypothesized that the extent to which this happens depends on the automaticity of reading processes, namely on the speed of print-to-sound conversion. We tested whether EVS is affected by another automaticity component – immunity from interference. To that end, we manipulated word familiarity (high-frequency, low-frequency, and pseudowords, PW) and word length as proxies of immunity from interference, and we used linear mixed effects models to measure the effects of both variables on the time interval at which readers do parallel processing by gazing at word N + 1 while not having articulated word N yet (offset EVS). Parallel processing was enhanced by automaticity, as shown by familiarity × length interactions on offset EVS, and it was impeded by lack of automaticity, as shown by the transformation of offset EVS into voice-eye span (voice ahead of the offset of the eyes) in PWs. The relation between parallel processing and automaticity was strengthened by the fact that offset EVS predicted reading velocity. Our findings contribute to understand how the offset EVS, an index that is obtained in oral reading, may tap into different components of automaticity that underlie reading ability, oral or silent. In addition, we compared the duration of the offset EVS with the average reference duration of stages in word production, and we saw that the offset EVS may accommodate for more than the articulatory programming stage of word N.

## Introduction

**FIGURE 1:**
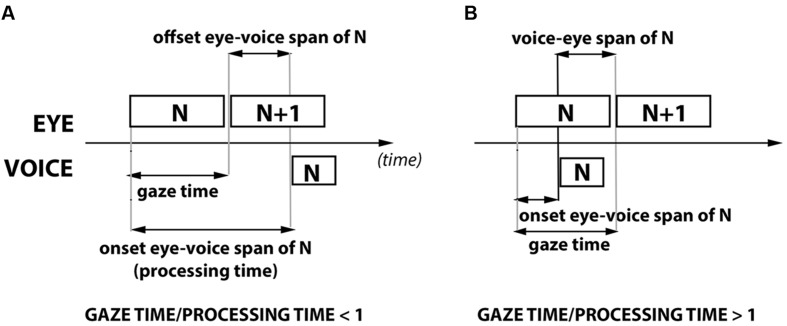
**Eye-Voice measures under two possible circumstances: **(A)** naming of N occurs while the reader views N + 1 (Eye-Voice Span) and **(B)** Naming of N occurs while the reader is still viewing N (Voice-Eye Span).** See text for more details (GT/PT, Gaze Time/Processing Time).

When readers name multiple items, the eye is usually ahead of the voice. This is known as eye-voice span (Inhoff et al., 2011; Pan et al., 2013; Laubrock and Kliegl, 2015) or eye-voice lead (De Luca et al., 2013). Eye-voice span (EVS) can be defined either in terms of space (the distance between the currently articulated item and the currently fixated one, *spatial EVS*), or in terms of time (how long it takes to articulate the item after having fixated it, *temporal EVS*). When EVS is defined in terms of time (**Figure 1**), a distinction is made between the time from the *onset of word fixation* to the onset of word naming (*onset EVS*), and the time from the *offset of word fixation* to the onset of word naming (*offset EVS*). The temporal onset EVS of word N is equivalent to the naming latency for that word. It encompasses all stages of word processing that take place before articulation, and may thus be referred to as the word’s processing time (**Figure 1**). The temporal offset EVS of word N refers to a shorter period. During this period, the reader gazes at word N + 1 while not yet having started to articulate N (**Figure 1A**). The temporal offset EVS is a particularly interesting period, in that it seems to signal the reader’s engagement in the parallel processing of N and N + 1, and thus some of her/his reading skills. Offset EVS is the focus of the present study, where we investigate the extent to which it depends on one of the components of automaticity - immunity to interference (Cohen et al., 1992; Moors and De Houwer, 2006; Moors, 2016).

Attention to EVS has resurged in the current decade, after a hiatus of nearly a century (see Buswell, 1921; Fairbanks, 1937). A major research goal has been to determine whether and how the length of EVS affects eye movements, and there has been agreement on the finding that eye movements on a word may be adjusted (the eyes may “wait for the voice”) for the sake of keeping a more or less constant EVS across the text (Inhoff et al., 2011; Laubrock and Kliegl, 2015). Since current models of eye movements in reading such as SWIFT (Engbert et al., 2005) and EZ-reader (Reichle et al., 2003) have been designed for silent reading, determining the influence of the eye-voice dynamics on eye movements is a means to expand these models to oral reading. A different and less emphasized research goal has focused on the reverse question, that is, what determines EVS itself. This is what we are concerned with in the present study, where we seek to better understand the meaning of the temporal offset EVS.

Why do readers gaze at a new word without having articulated the previous word? An available explanation for EVS is that the eyes tend to be ahead of the articulatory system because visual processing is faster than articulation (Laubrock and Kliegl, 2015). Although plausible, this approach seems insufficient once the influence of EVS on eye movements is considered: if the eyes can wait for the voice (Inhoff et al., 2011; Laubrock and Kliegl, 2015), why do the eyes go ahead? Specifically, why does the reader start gazing at word N + 1 before articulating N (offset EVS), if s/he seems able to delay the eyes and keep them on word N? The simplest answer seems to be that the lag between eyes and voice is useful. If the reader uses the initial gaze time on N + 1 to finish the processing of N (Jones et al., 2008, 2016; Protopapas et al., 2013; Laubrock and Kliegl, 2015) and does *parallel processing* of the two items, s/he saves time. If the eyes waited for articulation onset in order to move forward to the next word, the process would be less efficient. Of course, one may also admit that the reader already completed the processing of word N by the time he/she starts gazing at N + 1, in which case there would be no parallel processing. However, it is hard to explain why the reader would delay the articulation of N in that case. So, there seems to be no better explanation for the offset EVS period than the fact that parallel processing is unfolding, and the most likely scenario is that the reader is decoding item N + 1 at the same time that s/he plans the articulation of N (the last processing stage before articulation).

The notion that parallel processing takes place during offset EVS is not too controversial, but several questions remain unanswered. The first question concerns the cognitive constraints on parallel processing and, hence, on offset EVS. It has been suggested that the presence of an offset EVS period benefits from *automaticity* in reading (e.g., Laubrock and Kliegl, 2015). Automaticity is commonly approached as a multi-componential construct, in that it is defined by the combination of several features, or components (Cohen et al., 1992; Moors and De Houwer, 2006; Moors, 2016). Two of these components are processing speed (more automatic processes are faster; see Cohen et al., 1992; Moors and De Houwer, 2006; Moors, 2016) and release from attentional control, which in turn affords immunity from competing processes, or *immunity from interference* (Cohen et al., 1992). The relation between automaticity and EVS has been supported by findings that dyslexic subjects, who lack automaticity, show decreased EVS values compared to controls (De Luca et al., 2013), and the same goes for autistic subjects (Hogan-Brown et al., 2014). The idea that EVS reflects automaticity is also supported by findings that EVS predicts naming velocity for automatized processes such as digit naming, but not for less automatized processes such as dice naming (Pan et al., 2013). In these studies, the link between automaticity and EVS has been framed around the processing speed component of automaticity (Pan et al., 2013; Hogan-Brown et al., 2014; Laubrock and Kliegl, 2015): it has been argued that the speed (automaticity) of print-to-sound conversion is key to EVS. The potential role of the other automaticity component, immunity from interference, on EVS has remained unexplored. Nevertheless, immunity from interference is expected to facilitate the parallel processing of two adjacent words. If the processing of word N + 1, word N, or both, consumes few attentional resources, the processing of one word is immune to the competition of the other word, and the processing of several words may overlap in time (Protopapas et al., 2013), as it seems to occur during the offset EVS period. In order to examine how immunity from interference affects offset EVS, we used word familiarity as a proxy of this automaticity component.

The familiarity of a word is known to determine the relative activation of two different processes or routes, lexical and sublexical (Coltheart et al., 2001; Coltheart, 2006; Perry et al., 2007; Perry et al., 2010, 2013; Zorzi, 2010). High-frequency (HF) words (highly familiar) are expected to activate the lexical route more than low-frequency (LF; less familiar) ones and pseudowords (PW; totally unfamiliar), and PWs are expected to activate the sublexical route more than low- and HF words. Critically, the lexical route is known to be more automatic than the sublexical one, in the specific sense that it is immune to increases in memory load, while the sublexical route is not (Paap and Noel, 1991). Therefore, if immunity from interference determines offset EVS, we expect to see increased offset EVS values for HF words compared to the other classes. As a HF N + 1 word would require less attentional control and would be more immune to interference than an N + 1 PW, simultaneous (parallel) processing of N and N + 1 would be facilitated in the first case. Since lexical route processes are less dependent from word length than the grapheme-to-phoneme conversion processes of the sublexical route (Weekes, 1997; Rastle and Coltheart, 1998, 1999; Ziegler et al., 2001; Juphard et al., 2004; Zoccolotti et al., 2005; Barton et al., 2014), we also test for frequency × length interactions on offset EVS and we expect that the offset EVS of low familiarity words show increased length effects than the offset EVS of low-familiarity ones. To our knowledge, word familiarity effects on EVS have only been investigated by Halm et al. (2011), who found frequency effects on spatial EVS but not on temporal EVS. Since this paper is a very brief one, and the authors do not specify whether they measured the onset EVS or the offset EVS, uncertainty remains.

A second question pertains the type of relation between automaticity (in the sense of release from attentional control, leading to immunity from interference) and offset EVS. Possible evidence that automaticity *favors* the parallel processing taking place during offset EVS, i.e., that familiarity *modulates* offset EVS, does not lead to the obligatory conclusion that automaticity *is necessary* to parallel processing. In theory, parallel processing does not necessarily imply automaticity, and two scenarios may illustrate this possibility. One, the processes unfolding in parallel may depend on different cognitive subsystems which do not require the same attentional resources (Cohen et al., 1992). Two, the processes could require the same attentional resources, but the amount required by both may not exceed the available capacity. In these two scenarios, quantitative modulations of offset EVS by familiarity (shorter or longer EVSs) may still occur for several reasons. However, if automaticity is necessary, and the parallel processing of words is based on processes outside attentional control, lack of automaticity should *eliminate the possibility* of parallel processing, hence of offset EVS itself. Therefore, we posed the following question: under extreme decreases of automaticity, does a strict offset *eye-voice* span disappear, such that the eye “no longer leads” and the articulation of N begins while the reader is still gazing at N? Do low-automaticity settings, such as PWs, cause the eyes to remain on the word after naming onset? This scenario is portrayed in **Figure 1B**. For convenience, we named it simply *voice-eye span*, even though the voice onset is not ahead of the eyes in a straightforward manner, that is, the voice onset is not ahead of fixation onset, but ahead of fixation offset. In order to know the extent to which voice-eye span emerges (and parallel processing vanishes), we analyzed the distribution of offset EVS values for each familiarity x length condition, and we located the point at which offset EVS values become negative.

Whether automaticity is beneficial or necessary to offset EVS (and the possibility of parallel processing), offset EVS should predict reading velocity, since reading velocity itself depends on automaticity. A critical way of testing this would be examining whether the offset EVS of the experimental task predicts reading velocity in a concurrent task, since this would tap into readers’ automaticity skills in different contexts. Thus, in order to strengthen our analysis, we tested if offset EVS predicted reading velocity in the 3DM reading test (see Materials and Methods).

Finally, the notion that parallel processing takes place during offset EVS raises a third question – the question of *which processing stages of N* take place while readers gaze at N + 1. Two different perspectives are found in the literature. While Laubrock and Kliegl (2015) argued that word N enters the memory buffer (the offset EVS period) as a phonological form and parallel processing is restricted to motor (articulatory) planning, others (Jones et al., 2008, 2016) have claimed that previous processing stages of N, such as phonological processing, may develop during the offset EVS period. In order to shed some light on this, we explored the compatibility of the offset EVS in highly familiar words (highest EVS expected) with the estimated duration of the articulatory programming stage, which is around 150 ms (Indefrey, 2011). If we find offset EVS values considerably longer than 150 ms, this will suggest that processes other than motor planning (the last in the processing chain) may be part of the processing of N in parallel with N + 1.

Our approach is novel in two ways. First, unlike recent studies on EVS for text (Inhoff et al., 2011; De Luca et al., 2013; Laubrock and Kliegl, 2015), we present single words in blocked lists (HF, LF, and PWs in separate lists), rather than connected, sentence-like text. It is known that the combination of familiar and unfamiliar words (mixed lists) favors grapheme-to-phoneme conversion processes in familiar words (Lima and Castro, 2010) and thus decreases the familiarity-related contrast between lexical and sublexical processing. Since the effect of mixed lists is also expected in connected text, we used blocked lists to maximize such contrast and thus allow the emergence of extreme levels of automaticity (blocked HF words) and lack of automaticity (blocked PWs).

Additionally, we explore a novel approach to offset EVS, involving an EVS-related measure that we named *Gaze Time to Processing Time ratio* (GT/PT, **Figure 1**). GT/PT is obtained by dividing the gaze time on a word by the onset EVS (the processing time) of the same word. One advantage of GT/PT is that it is suitable for describing eye-voice span (gaze time shorter than processing time, **Figure 1A**) as well as voice-eye span (gaze time longer than processing time, **Figure 1B**). A second advantage of GT/PT is that it is a relative measure, describing the weight of different processing stages (gaze-dependent vs. gaze-independent) within the onset EVS (naming latency) period. A relative measure such as this is crucial to validate offset EVS results, since it describes *the contribution* of parallel processing to the complete processing (naming latency) of a given word, rather than just the duration of the parallel processing stage (offset EVS). Offset EVS (absolute) values may be misleading in the sense that differences in offset EVS between words do not necessarily mean different contributions of parallel processing. For instance, offset EVS values of 300 and 600 ms indicate equivalent contributions of parallel processing if naming latencies (onset EVS, or processing time) are 600 and 1200 ms, respectively (contribution of 50% in both). Conversely, it is possible that the offset EVS values of two words differ little (e.g., 300 ms vs. 350 ms), but such differences reflect important contrasts in the contribution of buffer-based processing (e.g., for processing times of 600 ms vs. 400 ms, respectively). In order to control for possible misleading effects of offset EVS (absolute) values, we performed the analysis of familiarity x length effects on both measures, and we compared the effects of both measures on scores of reading velocity.

## Materials and Methods

### Participants

Forty subjects volunteered to take part in the experiment, but four were excluded due to excessive eye artifacts. Thus, 36 Portuguese native-speakers (21 female; Mean age ±*SD* = 26 ± 5; Mean years of schooling ±*SD* = 15 ± 2) were included in the analysis. All had normal or corrected-to-normal vision. None had neurological problems or was taking drugs. Screening tests (QHL, Castro and Alves, 2005; 3DM, Reis et al., in preparation) showed no indications of reading disability. Participants signed informed consent, according to the declaration of Helsinki.

### Stimuli

**FIGURE 2:**
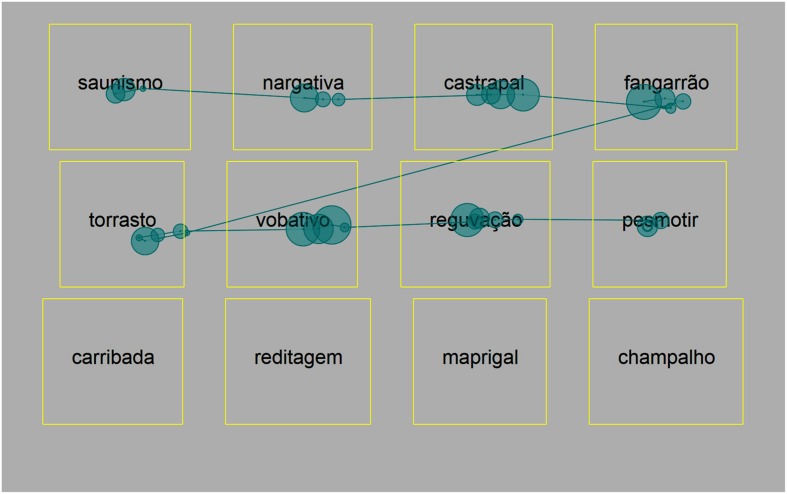
**Example list (long pseudowords).** Participants read the items in lines, as indicated by the example scanpath. Items in the first column, as well as the last item (“champalho”) did not enter the analysis. Rectangles around each item indicate the AOIs, which were not visible during the experiment.

We selected 80 HF and 80 LF words from CLUL database (Bacelar do Nascimento et al., 2000), which provides absolute frequency values found in a corpus of 16 210 438 words (see **Figure 2** to visualize log-transformed frequency values per class). We generated a set of 80 PWs (see Appendix). In each familiarity level, there were 40 short (4–5 letters; 30 regular and 10 irregular) and 40 long items (8–9 letters; 30 regular and 10 irregular). Short and long HF words did not differ in frequency (Means: short – 1339.90, long –1334.00), neither did LF words (short – 12.30, long – 12.98). The six familiarity × length levels were balanced for bigram frequency (Means: HF – short 60549, HF – long 64635, LF – short 64777, LF – long 63291, PW – short 62290, PW – long 61416) and neighborhood density (Mean 0.6 in all). In total, there were 240 (80 × 3) experimental stimuli organized into 30 lists for multiple-item presentation (see **Figure 2** for an example). The items in each list had the same level of familiarity, length and regularity status. Lists of long words or PWs comprised 12 items (3 rows × 4 columns), and lists of short words comprised 15 (3 rows × 5 columns). Filler items were included (filler words in word lists and filler PWs in PW lists), so as to avoid artifacts at critical positions (first column and last word slot of each list), and also to keep the number of items constant across lists. There were 156 filler items, summing up to 396 (240 + 196) stimuli.

The 3DM test, which we used for screening reading disability (see Participants), had a second purpose in our study: we also used it as a measure of individual reading velocity. We wanted to know whether offset EVS values for our experimental stimulus set could predict reading velocity in a concurrent test (see Statistical Analysis), so as to strengthen possible evidence that EVS taps into reading velocity (see Introduction). In the 3DM test, participants were presented with 75 LF words, 75 HF words and 75 PWs (none of these included in the eye-tracking experiment) for a fixed time interval. Their task was to name as many words or PWs as possible.

### Procedure

Participants were instructed to name the items, in rows, as accurately and fast as possible, while remaining still and avoiding blinking. They were asked to press the space bar of the computer keyboard at the end of each list. The 30 lists were randomly presented across subjects.

Eye movements were monocularly recorded at 1250 Hz with a tower-mounted SMI hi-speed eye tracking system^1^. Subjects placed their head on a chin rest and sat 80 cm away from the monitor. At this distance, the minimal inter-word spacing subtended 6.8° of the visual angle and was, thus, larger than parafoveal vision. Vocal responses were recorded with a Logitech webcam, synchronized with the eye-tracker as provided by SMI “Observation package” software. Subjects were first given practice trials. The recording session started with a thirteen-point calibration procedure, and tracking errors larger than 0.5° led to a new calibration.

### Data Pre-processing

Events were extracted with a high-speed algorithm, using a peak velocity threshold of 30° to identify saccades. Fixations shorter than 50 ms were rejected. Trials (lists) were visually inspected for artifacts, and those with more than 25% of signal loss were marked as contaminated trials. Subjects with more than 25% contaminated lists were excluded from the analysis (see Participants). Audio data were analyzed offline with Praat software^2^. Naming responses were classified for articulation accuracy (correct vs. misarticulated). Eye data per item × subject was scanned for blinks, lack of eye entry in the AOI (skipped item), and accidental eye entries at the onset of the list. Misarticulated items, as well as those containing any type of eye artifact (blink, skip or accidental entry) were removed from the analysis. Since the EVS is often readjusted by means of regressions (Inhoff et al., 2011), we excluded the items with second-pass reading from the analysis in order to keep the EVS uncontaminated from influences other than familiarity and length. After excluding misarticulations, eye artifacts, second-pass viewed items and outliers, we were left with 6734 data points for analyzing offset EVS, and 6504 data points (out of 8880) for Gaze Time to Processing Time ratio (GT/PT). Differences in the number of data points between the two variables were due to the exclusion of a different number of outliers in each.

Rectangular Areas Of Interest (AOIs) were placed around each word/PW (**Figure 2**) to compute first-pass gaze times and onset EVS per item x subject. *Onset EVS* was calculated as the interval between the first valid eye-entry on the item’s AOI and the naming (articulatory) onset. *Offset EVS* per item × subject was obtained by subtracting first-pass gaze time to onset EVS of item N. Positive offset EVS values indicate that the eyes are ahead of the voice (**Figure 1A**), and negative ones indicate the opposite (starting to name an item before the eyes move forward, see **Figure 1B**). Finally, *Gaze Time to Processing Time ratio* (GT/PT) values (first-pass gaze time/onset EVS) were obtained. Values larger than 1 follow positive offset EVSs (**Figure 1A**) and values smaller than 1 follow negative offset EVS values (Voice-Eye Span, **Figure 1B**). The distributions of offset EVS or GT/PT showed no marked deviations from normality.

### Statistical Analysis

We looked into descriptive statistics of offset EVS and GT/PT to determine if and when negative offset EVS and GT/PT values smaller than 1 (voice-eye span) would emerge. The mean and standard deviation of offset EVS and GT/PT for each stimulus class allowed us to estimate whether one standard deviation away from a positive offset-EVS mean (eye-voice span) would show negative values. This was complemented with percentile analyses, which specified, for each stimulus class, the percentile at which eye-voice span turned into voice-eye span. Mean offset EVS and GT/PT values were also used to investigate whether offset EVS periods might accommodate for processing stages other than motor programming (150 ms).

We used R (R Core Team, 2013) and *lme*4 (Bates et al., 2015b) to perform linear mixed effects analyses of the effects of frequency and length (fixed effects, with an interaction term) on offset EVS and GT/PT. As random effects, we had intercepts for subjects and items, but no by-subject or by-item random slopes. This was due to lack of convergence in random-slope models for our data, which seems to be in line with the attention that has been paid to the risk of overparametrization (Bates et al., 2015a). *P*-values were obtained by likelihood ratio tests of the full model with the effect/interaction in question against the model without that effect/interaction. Simple familiarity and length effects were tested against the intercept-only models, and familiarity × length interactions were tested against the model with both familiarity and length as fixed factors. To allow for these comparisons, models were fitted using the ordinary Maximum Likelihood (ML) criterion. We also followed the principle that, with a large sample size, absolute *t*-values larger than 2 indicate significant results at the 5% level (Baayen et al., 2008), and this principle was used to analyse frequency × length interactions (**Tables 2** and **3**).

We used similar procedures to test for offset EVS and GT/PT as predictors of reading velocity in the concurrent 3DM test. We modeled reading velocity with offset EVS or GT/PT as predictors, and compared the two models with the intercept-only model.

## Results

### Eye-Voice Span vs. Voice-Eye span

**FIGURE 3:**
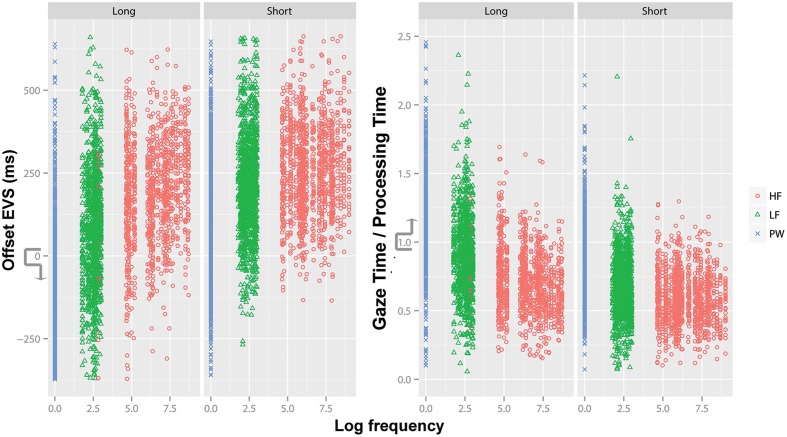
**Offset EVS **(left)** and Gaze Time/Processing Time **(right)** as a function of familiarity (*HF*, High-frequency, *LF*, Low frequency, *PW*, Pseudowords) and length (long vs. short).** Offset EVS values < 0 indicate Voice-Eye Span instead of Eye-Voice Span, and so do Gaze Time/Processing Time (GT/PT) > 1. Frequency values were log-transformed.

The mean values of offset EVS (**Figure 3**) for all items (*M* ±*SD*: 148 ± 187 ms) indicated that the readers’ eyes were, on average, fixating N + 1 (mean first-pass gaze time was 494 ms) when starting to name N. However, descriptive statistics for the six familiarity × length levels (**Table 1**) indicated a negative mean value for long PWs. Also, for LF long words and all PWs, negative offset EVSs started as soon as one standard deviation below the mean (e.g., for LF long, 81 – 174 is negative). GT/PT values (**Figure 3**) showed a similar picture, with a mean GT/PT > 1 for long PWs and GT/PTs > 1 starting one standard deviation above the mean in LF words and all PWs.

**Table 1:** Means (standard deviation) for offset EVS and GT/PT.

	HF		LF		PW	
			
	Short	Long	Short	Long^∗^	Short^∗^	Long^∗^
Offset EVS (ms)	258.9	191.4	210.2	80.9	122.7	-63.1
	(134.8)	(155.9)	(151.7)	(173.8)	(169.2)	(185)
GT/PT (First-pass/onset EVS)	0.59	0.71	0.68	0.89	0.82	1.15
	(0.18)	(0.23)	(0.22)	(0.28)	(0.27)	(0.36)


*^∗^Voice-eye span starting as soon as one standard deviation away from the mean.*

Percentile-based analyses indicated that GT/PTs > 1, or voice-eye spans, corresponded to percentiles 87, 74, 77 in HF short, HF long, LF short, and to percentiles 51, 59, 15 in LF long, PW short and PW long, respectively.

### Length of Offset EVS vs. Length of Motor Programming Stage

The average values of offset EVS for long and short HF words (191 and 259 ms), as well as for short LF words (210 ms, see **Table 1**), were large enough to accommodate for more than the average time of motor programming (150 ms).

### Familiarity and Length Effects on Offset EVS and VDPT

For both offset EVS and GT/PT the analysis of fixed factors (**Tables 2** and **3**) showed significant effects of familiarity, length, and a significant familiarity × length interaction, indicating that the effects of length increased as familiarity decreased. Concerning random factors, the variance arising from subjects was larger than from items in both cases.

**Table 2:** Predictors of offset EVS.

Fixed effects	Estimate	*SE*	*T*	Significance
Familiarity				χ^2(2)^ = 137.6, *p* < 0.001
LF-HF	-80.77	14.91-	-5.42^∗^	
PW-HF	-202.20	15.00-	-13.48^∗^	
Length (short–long)	134.80	13.76	9.80^∗^	χ^2(1)^ = 81.1, *p* < 0.001
Familiarity^∗^Length				χ^2(2)^ = 42.2, *p* < 0.001
LF(short–long) – HF	66.00	19.01	3.47^∗^	
(short–long)				
PW(short–long) – HF	130.53	19.27	6.77^∗^	
(short–long)				

**Random effects**		**Variance**	***SD***	

Item	Intercept	8323.00	91.23	
Subject	Intercept	6093.00	78.05	
Residuals		16684.00	129.17	


*Number of observations: 6734; Items: 240; Subjects: 36.*

**Table 3:** Predictors of Gaze Time/Processing Time.

Fixed effects	Estimate	*SE*	*T*	Significance
Familiarity				χ^2(2)^ = 147.2, *p* < 0.001
LF-HF	0.18608	0.02049	9.079^∗^	
PW-HF	0.44857	0.02132	21.038^∗^	
Length (short–long)	-0.22579	0.02253	-10.02^∗^	χ^2(1)^ = 84.5, *p* < 0.001
Familiarity^∗^Length				χ^2(2)^ = 44.3, *p* < 0.001
LF(short–long) – HF	-0.09339	0.02884	-3.24^∗^	
(short–long)				
PW(short–long) – HF	-0.20501	0.02948	-6.95^∗^	
(short–long)				

**Random effects**		**Variance**	***SD***	

Item	Intercept	0.00679	0.08242	
Subject	Intercept	0.01295	0.11382	
Residuals		0.04395	0.20965	


*Number of observations: 6504; Items: 240; Subjects: 36.*

### Offset EVS and GT/PT as Predictors of Reading Velocity in 3DM

**FIGURE 4:**
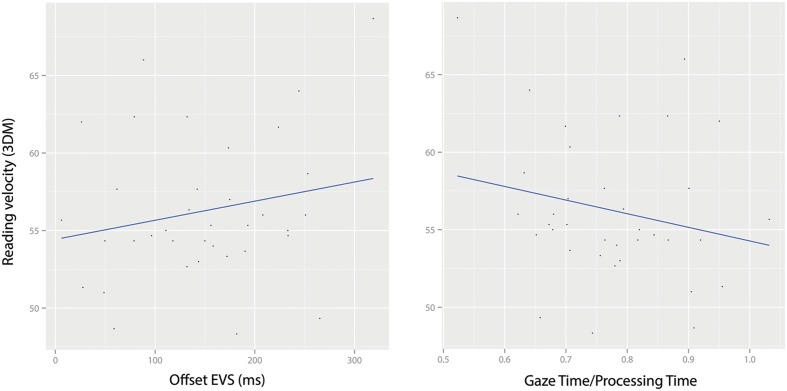
**Reading velocity (measured by 3DM, maximum 75 words) as a function of Offset EVS **(left)** and GT/PT **(right)**.** Subject means are plotted.

Both offset EVS and GT/PT predicted reading velocity scores. Reading velocity in 3DM increased as offset EVS in the experimental task increased [χ^2^(1) = 8.11, *p* = 0.004, see **Figure 4**], and it increased as GT/PT increased [χ^2^(1) = 7.38, *p* = 0.006].

## Discussion

Current approaches to the dynamics of eye and voice during oral reading suggest that the extent to which the eyes go ahead of the voice depends on the automaticity of the processes involved, automaticity referring to the speed of those processes. We have expanded the work on this hypothesis by focusing on a different component of automaticity – release from attentional control, leading to immunity from interference. This automaticity component is conceptually close to the parallel processing taking place during the offset eye-voice span period, and thus we investigated its role empirically, using word familiarity and its interactions with word length as proxies of immunity from interference. Our goal was threefold. First, we wanted to gather further evidence that automaticity leads to increased offset EVS values. Second, we wanted to determine if extreme decreases in automaticity eliminate parallel processing and transform eye-voice span into voice-eye span, so as to clarify if automaticity is necessary to offset EVS, rather than just beneficial. Third, we wanted to get preliminary information on whether the processing of word N in parallel with word N + 1 is limited to motor programming or, on the contrary, if it encompasses previous stages in the processing chain. We addressed these goals by investigating the effects of word familiarity and length on offset EVS as well as on an EVS-related measure that we named gaze time to processing time ratio (GT/PT).

Supporting our predictions, automaticity (immunity from interference) lengthened the parallel processing period corresponding to offset EVS. Less familiar words elicited shorter offset eye-voice span values (absolute measure) as well as stronger investment on gaze during word processing (longer GT/PT values – relative measure), compared to more familiar words. Due to the categorical approach we made, our analyses highlighted the effects of different *levels* of automaticity. From this viewpoint, we concluded that a PW (less familiar) requires, on average, longer offset EVSs than a HF word (more familiar). Nevertheless, gradient effects were also apparent: among HF words, words with the highest frequency values seemed to elicit the longest offset EVSs (see **Figure 3**), so it is highly likely that a continuous approach would also show significant effects. The effects of word length on offset EVS and GT/PT increased as familiarity decreased, signaling the interaction we predicted.

More than just beneficial, automaticity seems to be necessary to the parallel processing occurring during the offset EVS period. As familiarity decreased and length increased, a true eye-voice span vanished, and the reader started to name word N while still viewing it. Our data included many instances of voice-eye span, most of these found in LF words and PWs. In LF long words, data points that were one standard deviation above the mean represented voice-eye spans instead of eye-voice spans, and, in long PWs, even mean values did the same.

Finally, what is going on with word N while the eyes are ahead? Our findings are consistent with the possibility that the processing of N in parallel with N + 1 is not restricted to motor programming, the last stage before articulation. For HF (short and long) words and short LF words, we saw mean offset EVS values that accommodate for more than the average duration of articulatory programming, which is about 150 ms (Indefrey, 2011). For instance, according to our results, short HF words seem to allow both the syllabification (idem) and the articulatory programming of N in parallel with visual decoding of N + 1, that is, during the offset EVS period. From this viewpoint, the idea that a word must be phonologically coded by the time it ceases to be fixated in order to resist memory decay (Laubrock and Kliegl, 2015) does not seem to be supported, but we should be extremely cautious about this at least for two reasons. First, we are dealing with mere referential values; second, the fact that offset EVS exceeds the reference duration of motor programming does not necessarily mean that other processes are taking place, and the processing of N may be simply suspended for a fraction of the offset EVS period.

In the comparative analysis of offset EVS with GT/PT, both measures exhibited the expected familiarity x length interactive effects, and both predicted reading velocity in the expected direction (velocity increased with longer offset EVS and decreased with larger GT/PT). Therefore, our results for offset EVS seem valid enough. Although, in our case, offset EVS measures were not misleading since GT/PT indices did not change the picture, the concept of GT/PT expressed the observed negative offset EVS values (voice-eye span) in a simpler, less biased way. GT/PT values larger than 1 indicate that readers spend more time gazing at the word than the time needed to process it (begin its articulation). In contrast, the idea of a negative offset EVS is less transparent. Therefore, GT/PT seems to hold, at least, a conceptual advantage over offset EVS.

Our findings contributed to strengthen the link between offset EVS and the automaticity of reading, but the fact that we manipulated automaticity in an indirect manner, that is, using proxies (word familiarity and length), is one limitation. Direct manifestations of automatic processes may be captured with Stroop tasks (see Jones et al., 2016 for an example), which could be used in further studies to verify the relation between these processes and offset EVS.

In addition, our paradigm comprised a number of options that may have had a significant impact on our results. First, we chose to use lists of unconnected words because we wanted to potentiate familiarity effects. Finding out whether a different picture emerges (e.g., no voice-eye span) when using sentence-like materials that discard block effects and elicit semantic and syntactic integration, should stand as a next step in research. Second, we tried to eliminate parafoveal processing (Schotter et al., 2011) by controlling the inter-word space. The parafoveal processing that takes place when gazing at N + 1 (previewing N + 2) stands as an additional processing channel, and thus it is possible that there are less available resources for parallel processing during offset EVS when parafoveal processing is allowed.

The main contribution of our study was to strengthen the relation between offset EVS and automaticity in reading. Although we focused on measures that pertain to oral reading (offset EVS, GT/PT), the results of our study ultimately support the understanding of offset EVS (or its relative counterpart, GT/PT) as an index of automaticity, which underlies both oral and silent reading. Establishing offset EVS or GT/PT as indices of automaticity is an important step in clinical and experimental applications of the double-deficit hypothesis on dyslexia (Wolf and Bowers, 1999; Norton and Wolf, 2012), which proposed a distinction between phonological deficits and naming speed deficits in dyslexia cases. Lack of automaticity is a key feature of the naming-speed dyslexia type, which has been tapped with rapid automatized naming (RAN) tasks. Naming times have been used as indices of RAN performance, hence of automaticity. If offset EVS measures reflect automaticity, it may be helpful to add them when classifying dyslexia types.

Specifically, our study highlighted the relation between offset EVS and automaticity *viewed as immunity to interference*. Our findings are consistent with increasing evidence that dyslexic individuals – who typically show shorter EVSs – have problems in dealing with multiple presented items such as in RAN tasks (Jones et al., 2008, 2009, 2013; Zoccolotti et al., 2013), and they are particularly consistent with the interpretation that this is due to difficulties in managing between-item competing processes (e.g., processing one item while naming the previous one, and while previewing the next).

We wanted to test the effects of immunity to interference on offset EVS, and we used word familiarity as a proxy of immunity to interference. We did that based on Paap and Noel’s (1991) findings, which have not been consistently replicated (Pexma and Lupker, 1995). Therefore, there is the possibility that our assumption is incorrect, and that activating the lexical route by presenting HF words does not necessarily increase immunity from interference. Even if that is the case and we have not manipulated automaticity in our study, we are still left with evidence that the activation of the lexical route increases offset EVS. On the one hand, this may have implications for dual-route-based reading measures. Namely, it may afford measuring the reader’s reliance on the sublexical route using decreases in eye-voice span as an index. This would add to available behavioral (Crisp and Lambon Ralph, 2006) and eye-movement indices (e.g., Hawelka et al., 2010; Schattka et al., 2010; Rau et al., 2014). On the other hand, the fact that parallel processing is increased in the lexical route (longer EVS) raises new theoretical perspectives on dual-route approaches. It indicates that the lexical route affords a view-independent stage of word processing, while the sublexical route does not. If the reasons for this do not relate to different levels of automaticity in the two routes, they may, for instance, relate to increased levels of visual monitoring in the sublexical route, which, to our knowledge, is a new finding.

In our approach to the offset EVS, we put the emphasis on the extent to which it is a manifestation of parallel timelines of word processing, and many questions remain unanswered concerning these timelines. One question that is raised by our findings is why the eyes remain on the word during articulation in cases of voice-span, instead of moving on to the next word as soon as articulation begins. May articulation itself be dependent on gaze? For which purpose? Under which circumstances? We believe that this and other questions may strongly benefit from using methods of co-registration of eye-tracking and EEG in future research.

## Author Contributions

SS designed the experiment, collected data, analyzed data, and wrote the draft. LC collected data, analyzed data, and wrote the draft. AR and KP designed the experiment and revised the draft. LF designed the experiment, analyzed data and revised the draft. SS, AR, LC, KP, and LF did the final approval of the version to be published and showed their agreement to be accountable for all aspects of the work in ensuring that questions related to the accuracy or integrity of any part of the work are appropriately investigated and resolved.

## Conflict of Interest Statement

The authors declare that the research was conducted in the absence of any commercial or financial relationships that could be construed as a potential conflict of interest.

## Footnotes

1 www.smivision.com

2 http://www.fon.hum.uva.nl/praat/
