# Supplementary material for: When the Eyes No Longer Lead: Familiarity and Length Effects on Eye-Voice Span
Source: Front Psychol. 2016 Nov 2;7:1720. doi: 10.3389/fpsyg.2016.01720 (PMC5089997; doi:10.3389/fpsyg.2016.01720)
Supplement: Supplementary file 1 [file Table_1.docx]

Supplementary Material

When the Eyes No Longer Lead: Familiarity and Length Effects on Eye-Voice Span

Susana Silva*, Alexandra Reis, Luís Casaca, Karl Magnus Petersson, Luís Faísca

*** Correspondence:** Susana Silva, Neurocognition and Language Research Group, Center for Psychology at the University of Porto, Faculty of Psychology and Educational Sciences, University of Porto, Rua Alfredo Allen, 4200-135, Porto, Portugal, [zanasilva@gmail.com](mailto:zanasilva@gmail.com)

# Supplementary Data

Appendix - stimuli

| High Frequency (HF) | | Low Frequency (LF) | | Pseudoword (PW) | |
| --- | --- | --- | --- | --- | --- |
| SHORT | LONG | SHORT | LONG | SHORT | LONG |
| café | negativo | acne | taxativo | madé | tamarela |
| bebé | medicina | bidé | tagarela | umpo | cexarevo |
| júri | rigoroso | bule | carapuço | jule | jigoroso |
| táxi | cerâmica | caju | vocativo | esbo | vobativo |
| base | pesquisa | guru | cernelha | tafe | pesmilha |
| tabu | permitir | maná | masmorra | jufe | masquisa |
| maré | resposta | osga | campista | xevo | pesmotir |
| rede | encontro | orbe | propalar | guse | blascemo |
| nulo | proposta | tule | madrigal | réxa | profemir |
| bife | flexível | unto | blasfemo | xuna | maprigal |
| couve | habitação | pónei | carapinha | reipe | surailode |
| museu | revolução | naipe | bagaceira | naino | balicanha |
| peixe | narrativa | bouça | fuzileiro | reiça | reguvação |
| gesso | vacinação | rojão | serradura | beite | cabeleida |
| cupão | cabeceira | gibão | barricada | faute | sarrivada |
| beijo | televisão | quedo | pegureiro | mubão | balaceira |
| reino | sucessivo | sifão | saraivada | gipão | capoceiro |
| roupa | terramoto | fauno | cachalote | reife | catapinha |
| tarde | diferença | dedal | indigesto | vesna | nargativa |
| papel | dirigente | vesgo | cabotagem | vorfa | reditagem |
| filme | ginástica | jaspe | cavalgada | moliz | caxaltiça |
| total | ginástica | lorpa | repetente | begor | pelorente |
| líder | eleitoral | ginja | campesino | férus | repelanto |
| dólar | casamento | sapal | dirigismo | tolim | giritento |
| feliz | municipal | lúpus | petulante | vimol | dipetendo |
| vírus | resultado | móbil | pilotagem | sárus | renimenca |
| rival | chanceler | bemol | carrascão | mópis | chuvercar |
| vital | calcanhar | bilro | bairrista | pamur | sustincar |
| lápis | suspensão | selim | chamuscar | sezir | caquinhar |
| luzir | banqueiro | bílis | charneira | xorba | fangarrão |
| civil | linguagem | fémur | churrasco | ripel | carfascão |
| arroz | distinção | lince | carrossel | sadal | pegulante |
| nuvem | mesquinho | furna | fanfarrão | pazol | jesquinho |
| grupo | finlandês | lugre | casquilho | zegre | mistinção |
| fluxo | charneira | sabre | metralhar | flubo | proguiste |
| negro | orquestra | zebra | doutrinal | labro | castrapal |
| lapso | principal | tripé | chanfrado | rubre | prolhando |
| rubro | proporção | brejo | castrense | glopo | disgrital |
| lebre | brilhante | greda | flautista | gnoxo | mepralhar |
| globo | distrital | gnomo | droguista | brepo | casprense |

**
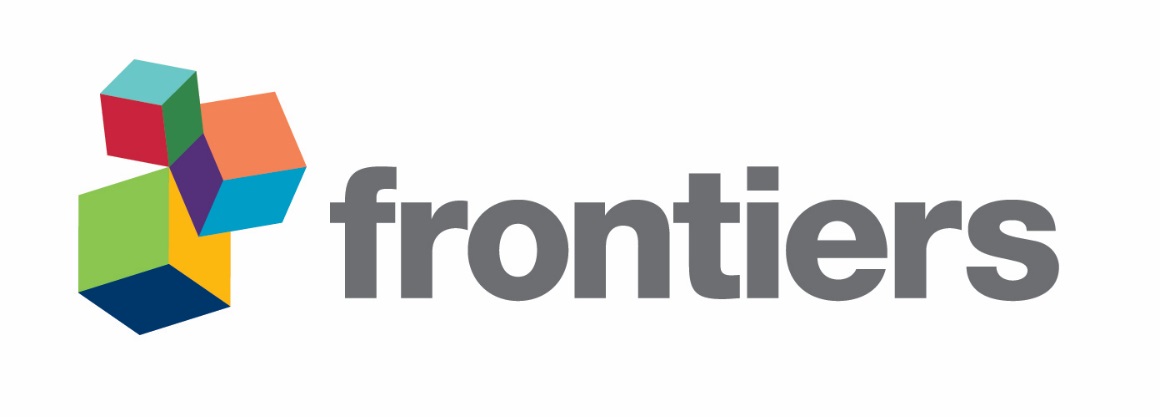
**
